# Abscisic Acid Mediates Drought and Salt Stress Responses in *Vitis vinifera*—A Review

**DOI:** 10.3390/ijms21228648

**Published:** 2020-11-17

**Authors:** Daniel Marusig, Sergio Tombesi

**Affiliations:** Dipartimento di Scienze delle Produzioni Vegetali Sostenibili, Università Cattolica del Sacro Cuore, 29122 Piacenza, Italy; daniel.marusig@unicatt.it

**Keywords:** ABA, grapevine, stomata, drought, metabolism, carbohydrates, salinity

## Abstract

The foreseen increase in evaporative demand and reduction in rainfall occurrence are expected to stress the abiotic constrains of drought and salt concentration in soil. The intensification of abiotic stresses coupled with the progressive depletion in water pools is a major concern especially in viticulture, as most vineyards rely on water provided by rainfall. Because its economical relevance and its use as a model species for the study of abiotic stress effect on perennial plants, a significant amount of literature has focused on *Vitis vinifera*, assessing the physiological mechanisms occurring under stress. Despite the complexity of the stress-resistance strategy of grapevine, the ensemble of phenomena involved seems to be regulated by the key hormone abscisic acid (ABA). This review aims at summarizing our knowledge on the role of ABA in mediating mechanisms whereby grapevine copes with abiotic stresses and to highlight aspects that deserve more attention in future research.

## 1. Introduction

Climate change is expected to have negative impacts on the socioeconomic system [1]. Despite the discrepancy between the projected scenarios, even the most optimistic models foresee an increase in the occurrence and duration of anomalous droughts, especially in the Mediterranean-climate regions, where water sources will be increasingly scarce [2]. These threats are of major concern to agriculture and in particular to viticulture, being the one of the most profitable crops in these regions [3]. In 2016, less than 10% of vineyards in the European Union were irrigated, even if they accounted for approximatively 60% of world’s grape production [3]. Thanks to the lately moderation of restrictions imposed by law, in many states the use of irrigation has increased (e.g., in 2018, the irrigated vineyards’ area in Spain exceeded 30%) [4,5]. The deleterious effects of climate change will negatively affect water sources extent and quality, and the solely irrigation is not a sustainable and sufficient strategy to counteract the expected impacts on grape production [3]. In order to cope with these constraints, a general improvement in viticulture techniques is needed and the achievement of this goal requires a better knowledge of grapevine physiology under stress conditions.

*Vitis vinifera* is a Mediterranean vine [6] mostly cultivated in Mediterranean-like areas, and in particularly arid environments like Karst [7]. Grapevine is adapted to cope with drought conditions and some common cultivation techniques are based on imposing moderate soil water deficits, in order to improve the quality of berries, minimize the yield reduction and favor the production of flavonoids, sugars, polyphenols, and carotenoids [5].

Further to its economical relevance, *V. vinifera* is a model species in drought-response investigation [8]. The drought-tolerance strategy of grapevine consists in an ensemble of interactions between morphological/structural traits and a pronounced control of water loss by stomatal regulation [5]. The latter one is mostly mediated by hormonal regulation, and a pivotal role in this process is played by the abscisic acid (ABA) [9].

ABA is a ubiquitous hormone, which has been discovered to modulate physiological responses among all the kingdoms of life [10]. In vascular plants, it regulates a multitude of physiological processes like seed and bud dormancy [11], cambium activity [12], organs development [13], and fruit ripening [14]. Yet, it is involved in mediating physiological responses to stressful conditions, especially excessive temperature, salinity, and drought [15,16,17,18]. ABA has been mainly investigated as it promotes the loss of turgor pressure in guard cells triggering stomatal closure [9], however, an increasing amount of evidences, also based on *V. vinifera*, address to ABA a central role in other fundamental processes as the hydraulic response to prolonged drought conditions [19], the regulation of carbohydrate metabolism during recovery [20], and the exclusion of excessive salts dissolved in the soil solution [21].

Since the socioeconomic importance of *V. vinifera* and the increasing number of studies asserting ABA to be a major factor in regulating a so far underestimated amount of physiological responses, the aims of this review are: (i) to provide an exhaustive overview on the role of ABA in *V. vinifera* as a key hormone involved in regulating the mechanisms for coping with the major threats caused by climate change and (ii) highlight the main issues that deserve further investigation.

## 2. ABA Biosynthesis and Translocation

**Figure 1:**
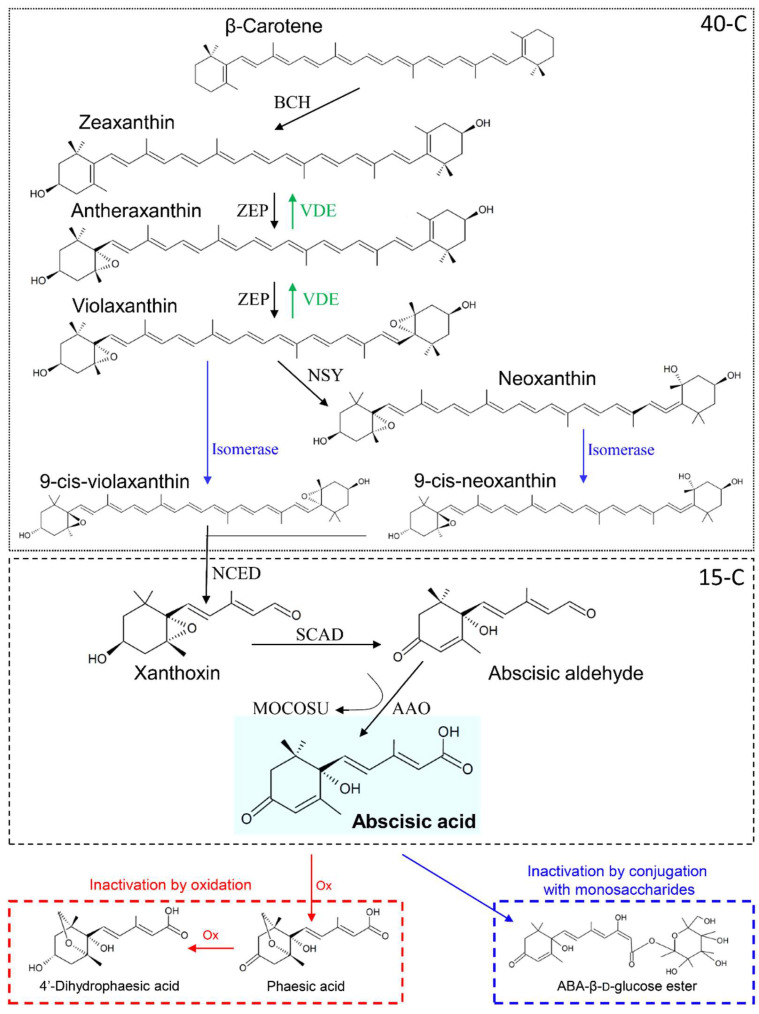
Abscisic acid biosynthesis and metabolism. In the first step, β-carotene is di-hydroxylated by β-carotene hydroxilase (BCH) proteins to produce the transisomer zeaxanthin. Hence, zeaxanthin is epoxidated by zeaxanthin oxidase (ZEP) to antheraxanthin and, then, to violaxanthin. ZEP-mediated reactions can be reversed by violaxanthin de-epoxidase (VDE). Violaxanthin can be transformed into neoxanthin by neoxanthin synthase (NSY) and both violaxanthin and neoxanthin are converted in the respective 9-cis-isomer by isomerase catalysts. The 15-carbons apocarotenoid sesquiterpenoid xanthoxin is then produced by cis-xanthophylls cleavage, whose reaction is catalyzed by 9-cis-epoxycarotenoid dioxygenase (NCED). Subsequently, xanthoxin is oxidized to abscisic aldehyde by short-chain alcohol dehydrogenase (SCAD), and finally abscisic acid (ABA) is produced by oxidation of abscisic aldehyde through the combined action of ABA-aldehyde oxidase (AAO) and a molybdenum cofactor sulfurase (MOCOSU). ABA can be inactivated by oxidation or by conjugation with monosaccharides. In the first way, ABA is oxidized (Ox) at first to phaseic acid and then to 4′-dihydrophaseic acid. In the second one, ABA is conjugated with glucose to produce ABA-β-D-glucose ester. Based on [9].

ABA is a 15-carbons isoprenoid, derived from the metabolism of β-carotene (40 carbons), originated from the methylerythritol 4-phosphate (MEP) pathway [22]. The β-carotene to ABA-biosynthetic pathway is composed by many steps, and it is schematized in Figure 1. In the first part of the pathway (40-carbons), the β-carotene is converted into the isomer zeaxanthin (Zx), which is metabolized through an ensemble of conversions called the “xanthophyll cycle,” having final product violaxanthin (Vx) and neoxanthin (Nx). Both Vx and Nx can be converted into the respective 9-cis-isomers, from which cleavage, the sequisterpenoid xanthoxin (Xx) is produced. At this step, the 15-carbons pathway starts leading to the synthesis of ABA [9].

Being ABA produced by cleavage of carotenoids, research has initially focused on investigating the leaf as main site of its biosynthesis [23,24,25]. In 1974, Loveys and Kriedemann [23] investigated the role of leaf-produced-ABA causing stomatal closure in *V. vinifera* cv. Cabernet Sauvignon, by measuring ABA concentration ([ABA]) in detached leaves. Their results assessed significant increases in [ABA] and stomatal resistance, supporting the hypothesis of endogenous ABA triggering stomatal closure. A few years later, the same authors [24] measured [ABA] in extracts of *Spinacea oleracea* leaves, speculating that most of the ABA is contained in the chloroplasts, and it is present also in nonstressed leaves. In contrast to these results, evidences on *Vitis vinifera* cv. Riesling and Silvaner pointed out that [ABA] in xylem sap ([ABA]_xy_) was related to stomatal conductance (g_s_) regulation, suggesting that ABA may be translocated to the roots through the phloem and back to the shoot through the xylem, guaranteeing a continuous supply to leaves [25].

Since ABA biosynthesis in the leaf would require stress-induced stimuli (e.g., loss of turgor and shrinkage) [26], root system was proposed as a primary site for ABA biosynthesis [27,28]. In 1987, Zhang and Davies [27] observed the production of ABA in detached root tips of *Pisum sativum* and *Commelina communis*, providing one of the first strong evidences supporting the hypothesis that ABA is synthesized in root tips and transported from roots to shoot via the transpiration stream. Soar et al. [29] reported that in Shiraz vines, [ABA]_xy_ increased as drought was more intense. The increase in [ABA]_xy_ changed according to the different rootstock, suggesting that the different root system may affect the ABA production and delivery [29]. These conclusions have been supported also by gene-expression analyses. In 2013, Speirs et al. [30] measured [ABA] and expression of the ABA biosynthesis genes VviNCED1 and VviNCED2, in Cabernet Sauvignon grapevine roots and leaves. As drought stress increased, [ABA] strongly increased in roots, xylem, and leaves, especially in the latter. Nevertheless, gene expression remained stable in leaves, but remarkably increased in roots.

These findings led to the development of an irrigation technique, named “Partial Root Drying (PRD),” aimed at optimizing grapevine water use, in order to reduce irrigation and improve berry quality [31,32,33,34]. This objective is pursued inducing ABA production in the root system, hence limiting water loss by triggering stomatal closure [31]. From a technical point of view, while water supply is provided by watering part of the root system, in the remaining part, irrigation is withdrawn, in order to stimulate ABA production [35]. Hence, ABA is transported through the xylem to the leaves by the driving force generated from transpiration (see Section 3) [17]. In order to guarantee a stable production and translocation of ABA, the wet and dry parts of the roots are weekly switched [17,36]. In 2007, Poni et al. [32] evaluated the physiological response and the yield-quality performance of Sangiovese grapevines under PRD irrigation regime. Their results highlighted that under PRD, the water use efficiency was improved as g_s_ was strongly reduced, while the decrease in photosynthetic assimilation (A) was more limited. If compared to the well-watered (WW) treatment, the PRD had also positive effects on the control of vigor, it improved the berry quality and it did not cause variations in yield [32]. Romero et al. [34] investigated physiological responses of Monastrell grapevines under different irrigation regimes, for 4 years. Under PRD, [ABA]_xy_ significantly increased, following the trend of depletion in soil water availability. Moreover, under PRD, vines developed a deeper root system and maintained a better water status than those irrigated with the same water volumes but with regulated deficit irrigation (RDI), where water was totally withdrawn for a limited period [34]. Even an RDI strategy would set a portion of the root system that insists into a dry soil volume and stimulate ABA production. However, [ABA]_xy_ seems to depend on the volumetric soil water content of both wet and dry sides [16].

The hypothesis of ABA being mainly synthetized in the root system has been commonly accepted [37], and it is still supported by more recent research [38]. Despite that, an overwhelming body of literature has been supporting the hypothesis that drought-induced stomatal closure is also mediated by in-site produced ABA into the leaf [39,40,41,42,43,44]. A first issue is represented by precursors abundancy: the ABA biosynthetic pathway is based on carotenoid, whose accumulation depends on the ability of the plastid to sequester them in specific sinks [45]. Due to the presence of chloroplasts and chromoplasts, this is ordinary in leaves. On the contrary, in roots plastids are mainly proplastids and leucoplasts, which cannot accumulate carotenoids [46], and a root-to-shoot ABA supply pathway seems unlikely [41]. In 2009, Ikegami et al. [40] observed rising [ABA] in roots and leaves of *Arabidopsis thaliana* under drought. However, when the same measurements were replicated on the same tissues detached from the plant, [ABA] increased only in leaves. These observations have been strongly supported also by gene expression analyses [39,47] and solid evidences claim the mesophyll being the main site of ABA biosynthesis in leaf [42,43]. In 2006, Soar et al. [39] measured the diurnal variation of [ABA]_xy_ and expression of ABA-biosynthetic genes in Grenache and Shiraz grapevines. They observed that at midday, under high evaporative demand, [ABA] and the expression of VviNCED1 and VviZEP increased in leaf and remained stable in roots [39]. McAdam and Brodribb [42] measured [ABA] in bench-dried leaves of five different species (one angiosperm and four gymnosperms), which leaf anatomy allowed to isolate mesophyll, vascular tissue, and stomata. They observed that drought-induced production of ABA mainly occurs in the mesophyll [42], hence supporting the hypothesis that mesophyll shrinkage, due to cell volume decline, may trigger the whole process [43].

This topic is still debated, but a few researches aimed at investigating leaf as the biosynthetic source of ABA in grapevine. Indeed, in grapevine, the investigation of stress response was more focused on cultivar [48,49], scion–rootstock interaction [50], observational scale [51], geographical area [49], acclimation, and experienced drought stress [20]. However, the localization of the response to stress mediated by ABA represent an important information for the application of specific viticulture techniques such as irrigation.

## 3. ABA Role in Regulating Stomatal Closure

Water uptake and transport through the xylem conduits are regulated by a tension-cohesion mechanism [52]. Water loss at the leaf level leads to a decrease in leaf water potential (Ψ_leaf_). Hence a dynamic water potential gradient is established, which triggers water flow from roots to leaves [52]. As drought persists and water availability accordingly decreases, tension in the xylem conduits increases and the water transport is weighted down by the occurrence of embolism events [53]. Without restoration, the presence of emboli in the xylem conduits breaks the continuity of the water column and prevents water transport through the xylem, leading in the worst cases to hydraulic failure and plant death [54].

**Figure 2:**
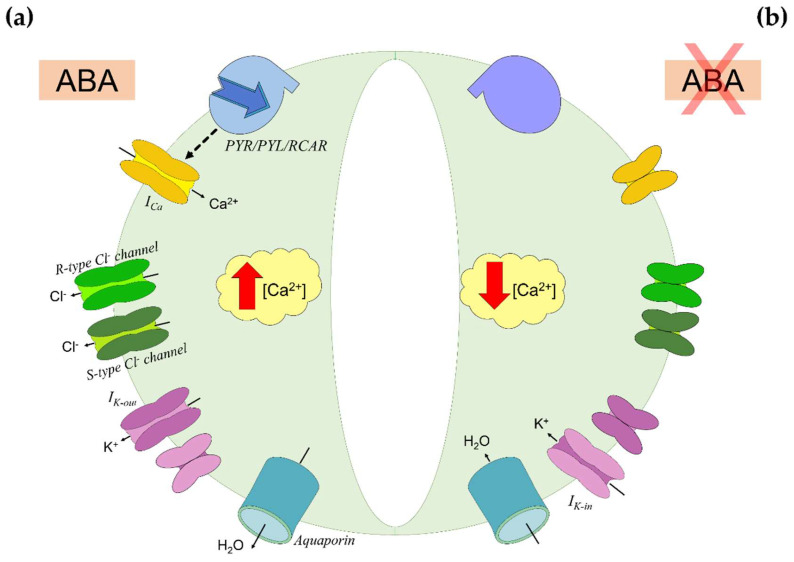
Abscisic acid (ABA) signaling in guard cells. (**a**) ABA inducing stomatal closure. ABA binds to PYR/PYL/RCAR receptors on the guard cells membrane and triggers the accumulation in cytosol of Ca^2+^ by activation of Ca^2+^ channels (I_Ca_). Under elevated Ca^2+^ concentration ([Ca^2+^]), the cell-efflux of Cl^−^ is enhanced. This efflux is mediated by rapid transient (R-type) and slow-activating sustained (S-type) Cl^−^ channels, and it causes plasma membrane depolarization. Thence, the K^+^ uptake is downregulated by inward-rectifying K^+^ channels (I_k-in_) activity, while the K^+^ efflux is promoted through outward-rectifying K^+^ channels (I_k-out_). The overall reduction in ions content triggers water efflux through aquaporins by osmosis, causing loss of turgor in guard cells and stomatal closure. (**b**) Stomatal reopening by ABA inactivation. As ABA does not bind further to PYR/PYL/RCAR receptors, Ca^2+^ accumulation ceases. The osmotic balance is restored by K^+^ uptake through I_k-in_, promoting water uptake and reacquiring turgidity. Based on [56].

Plants are able to modulate water loss through stomatal closure, a process influenced by an ensemble of environmental (e.g., light intensity), hydraulic (e.g., hydraulic conductance), and endogenous (e.g., hormones) factors [55]. The most important factor between the endogenous ones is ABA, which triggers stomatal closure by inducing loss of turgor in guard cells and prevents stomata premature reopening (Figure 2) [56]. When ABA binds to receptors on the guard cells membrane, it triggers the accumulation in cytosol of Ca^2+^ and the efflux of Cl^−^ and K^+^ ions [57]. Osmolytes displacement causes plasma membrane depolarization and the overall reduction in ion content triggers water efflux by osmosis, causing loss of turgor and stomatal closure [9]. This status persists as long as ABA gets inactivated by oxidation or conjugation with monosaccharides (Figure 1). Then, Ca^2+^ concentration ([Ca^2+^]) decreases, and the osmotic balance gets restored by K^+^ uptake [56,57].

**Figure 3:**
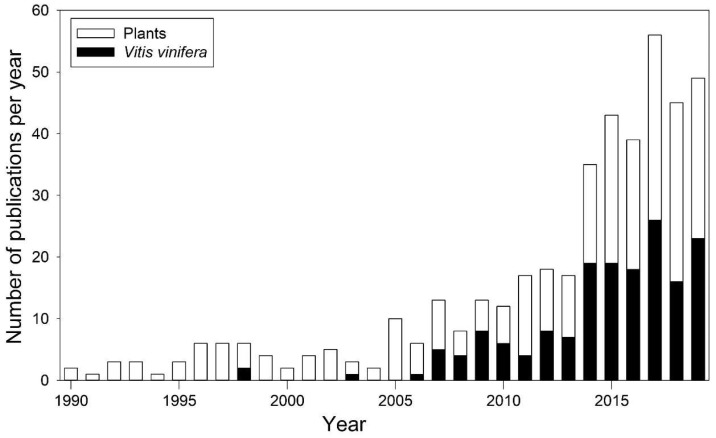
Number of publications dealing with iso-/anisohydry from 1990 to 2019, for all plant species (white bars) and *Vitis vinifera* (black bars) only. Data collected from the Scopus database (https://www.scopus.com/), according to the method of [60].

ABA role in stomatal regulation has been particularly studied in grapevine, in order to optimize irrigation techniques and genotype-specific response to drought. Irrigation techniques aim at maintaining plant water status and at maximizing plant water use efficiency (WUE), expressed as the ratio between photosynthetic CO_2_ assimilation (A) and water loss by transpiration (E) [48]. In grapevine, cultivars differently regulate water loss as they differ in terms of stomatal response under high evaporative demand [5]. This behavior has been addressed using the concept of iso-/anisohydry [58] that generally refers to the daily variation of the difference between Ψ_leaf_ and soil water potential (Ψ_soil_). Specifically, isohydric cultivars maintain a more constant water status through preventive stomata closure, while anisohydric cultivars keep stomata open until more negative Ψ_leaf_ values [59]. Isohydry has been associated to differences in the ABA signaling process [58]. Since *V. vinifera* displayed a high intraspecific plasticity in anisohydric behavior, it became one of the most investigated species (Figure 3) [60]. In 2001, Bota et al. [48] investigated gas exchange and hydraulic responses to drought in 22 grapevine cultivars. Despite most of the cultivars belonged to the same region, as drought persisted, they displayed a remarkably divergent iso-/anisohydric behavior. Differences in anisohydricity have also been observed by Coupel-Ledru et al. [61], which assessed a divergent stomatal response to drought, even in pseudo-F_1_ progeny of a reciprocal cross between the Syrah and Grenache cultivars.

The intraspecific divergence in stomatal behavior, may be related to a genetic difference in ABA production [58]. Nevertheless, an increasing amount of evidences has highlighted that many other factors are involved in stomatal regulation [5]. Indeed, studies speculating of ABA being the pivotal driver of drought-response have mostly investigated grapevine physiology under severe water stress. However, differences in isohydric behavior can be acknowledged at moderate water stress [49]. Recently, Levin et al. [49] characterized the response of g_s_ along a wide range of Ψ_leaf_ in 17 grapevine cultivars; they showed that cultivars differed in g_s_ response only under moderate stress, while under well-watered conditions and severe stress, the intraspecific difference is not appreciable.

As abovementioned, under water shortage, tension in xylem increases inducing the occurrence of embolism events, occluding the conduits and reducing the hydraulic conductivity (K_xy_) [54]. It has been suggested that cavitation occurrence may act as a hydraulic signal, triggering the stomatal response [62]. This mechanism has been observed on crops [19], herbs [63], and trees [64], also being common between species with contrasting origin and anatomy [62]. Based on this hypothesis, stomatal control strategy of plants should depend on its vulnerability, generally expressed as the water potential at 50% loss of conductivity. This speculation has been strongly supported on a wide range of species [65] and in grapevine [66], however, the debate is still open as some evidences support a more complex interaction [67], also claiming that stomatal closure may precede emboli formation [68].

**Figure 4:**
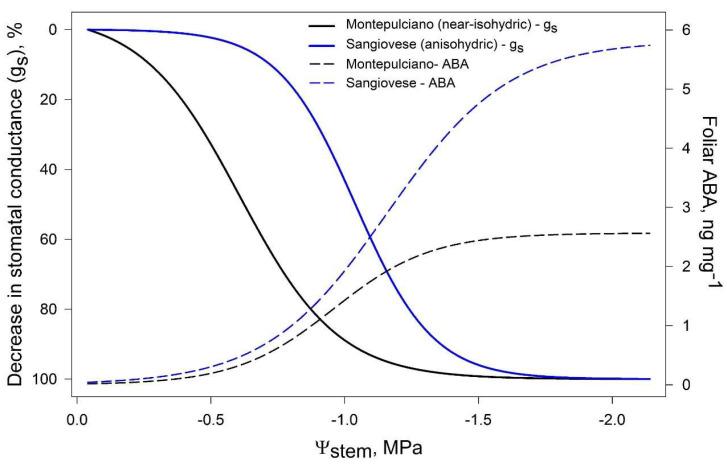
Modelled dynamic of stomatal conductance (g_s_) and foliar ABA concentration in a near-isohydric cv (Montepulciano) and an anisohydric cv (Sangiovese). Elaboration on data by [19].

McAdam and Brodribb [69], in a study on *Metasequoia glyptostroboides*, suggested that stomatal closure may be triggered by passive hydraulic signals, while active ABA-mediated regulation occurs under long-term drought. In grapevine, Tombesi et al. [19] observed that foliar [ABA] significantly increased after g_s_ was already low, suggesting that stomata closure is regulated differently according to the intensity of drought (Figure 4). As water shortage begins, stomatal closure is likely triggered by hydraulic signals, while long-term ABA maintains turgor loss in guard cells, preventing premature reopening [19]. Similar results have been recently obtained on Merlot grapevines by Degu et al. [70] and may support the evidences of isohydric and anisohydric cultivars displaying different ABA/hydraulic signals stomatal regulation [71].

Interestingly, some studies assessed a relation between ABA and hydraulic properties, suggesting that ABA may have an indirect effect on water loss management [72,73]. Soar et al. [39] observed that differences in stomatal responses between Grenache (near-isohydric) and Shiraz (near-anisohydric) were related to [ABA]_xy_. They suggest that the degree of isohydricity may be related to differences in hydraulic properties, which depend on the interaction between ABA and other factors, in particular, aquaporins. In this way, high [ABA] inhibits aquaporins expression, downregulating K_xy_ and inducing stomatal closure [74]. Recently, Dayer et al. [73] investigated the coordination between ABA, aquaporins expression, and hydraulics of root and shoot in Grenache (near-isohydric) and Syrah (near-anisohydric) grapevines under mild water stress. Their results highlighted that even under mild water stress, ABA production causes downregulation of aquaporins expression in the leaf, in order to prevent water loss. The entity of this phenomenon was different according to the cultivar, suggesting that less anisohydric cultivars are more sensible to ABA [73]. Despite these evidences, our knowledge on this topic is still scarce, especially on grapevine [75]. Future research is still needed, in order to better understand the complex relations occurring between ABA and hydraulic regulation, which occurs with a high intraspecific variability.

## 4. ABA Role in Carbohydrates Mobilization

Threats related to the foreseen increase in occurrence and duration of anomalous dry conditions due to climate change are expected to affect the vegetation in multiple ways. As far as drought persists, plants are forced to keep stomata closed for prolonged periods, limiting the carbon uptake [76]. Over prolonged drought, stomatal limitation may affect the balance between carbon supply from photosynthesis and consumption, leading to carbon starvation and consequent lack of energy to drive metabolism and repair damaged photosystems [76]. Thus, after prolonged drought, plants depleted in carbohydrates are more likely to incur into hydraulic failure, due to the inability in recovering damage, even after stress relief [77].

Recent evidences on poplar, highlighted that recovery of embolized conduits is a spatially coordinated and energy-demanding process, which requires the active translocation of sugars and other resources from the symplast to the apoplast of parenchyma cells [78]. These results confirm previous observations on grapevine [79] and support the studies speculating that different grapevines cultivars recover differently according to a divergent ability in translocating and utilizing nonstructural carbohydrates (NSCs) [20].

Since ABA is a key factor in modulating the starch-to-sugars pathway, by upregulating carbohydrate metabolism’s enzymes (e.g., β-amylase and vacuolar invertase) [80,81], some studies have suggested that it may play a pivotal role in carbohydrate mobilization response during recovery [81,82,83,84]. Secchi et al. [82] applied exogenous ABA to ABA-deficient mutants of *Lycopersicon esculentum,* showing that increasing [ABA] favors embolized-vessels refilling. Differences in refilling were also correlated with petioles starch content, supporting the hypothesis of ABA being able to trigger sugars translocation for damage repairing. A detailed investigation on ABA role in sugars metabolism and mobilization has been recently conducted on *Populus nigra* by Brunetti et al. [83]. They observed that at recovery, bark-stocked starch is rapidly converted to soluble sugars, which are then translocated in the wood. Both starch depletion and soluble sugars increases have been correlated to [ABA], suggesting ABA being the trigger [83]. In grapevine, Perrone et al. [6] evaluated concurrently physiological response, ABA variation, and gene expression, in leaf petioles during water stress and subsequent recovery. Since the coordinated increment in [ABA] and expression of genes related to secondary metabolism during recovery, it is likely that ABA may play a pivotal role in coordinating the carbohydrates mobilization.

These studies increased our knowledge about the starch-to-NSCs conversion and subsequent mobilization during post-drought hydraulic recovery [85]. Furthermore, these observations may suggest a possible role of ABA in mediating processes related to carbohydrates metabolism where its role has not been considered so far. A recent study [20] pointed out that under drought, different grapevine cultivars display a contrasting pattern of NSCs utilization. The anisohydric Shiraz delayed NSCs consumption, and it implemented remarkable anatomical adjustments (decreased the size of xylem conduits). Conversely, in the near-isohydric Cabernet Sauvignon, stress response was based on an earlier starch depletion and NSCs mobilization [20].

The iso-/anisohydric classification has been redefined multiple times, the species/cultivar behaviors are distributed along a continuum rather than being dichotomous, and its variation depends on the plant interaction with the environment [60,65]. Nevertheless, the concept itself is useful to define the plant-specific stress management under long term [59]. Different grapevine cultivars have been sorted according to their iso-/anisohydric behavior; however, it has been observed that under different treatment/conditions, the same cultivar can display different iso/anisohydric behavior [51,60,86]. Since the increasing amount of evidences have linked ABA to a long-term regulation mechanism, and since it has been proved that differences in grapevine intraspecific anisohydricity have been linked to a variation in ABA biosynthesis [87], it seems likely that ABA may coordinate the ensemble of phenomena involved in stress response. Further investigation on the actual role of ABA is still needed, especially on poorly understood adaptation mechanisms as “stress memory” [88,89], which has also been related to modulations in carbohydrate metabolism [89,90,91] and have also been observed in grapevine [20,92].

## 5. ABA in Salt-Stress Response

Foreseen effects of climate change are expected both to increase the water consumption demand in agriculture and to decrease the abundance of water sources [2]. Therefore, the use of wastewaters for crops irrigation has been purposed [3]. Besides the reduction in freshwater use, the application of this strategy has led to many benefits as the improvement of nutrients recycling, the minimization of pollutants’ discharge into waterways, and the increase in plant growth, photosynthesis activity, and carbohydrates production [3,9]. However, over long term, this strategy may be deleterious, because of the elevated salts content in wastewaters [93]. Excessive salts concentration in the circulating solution in soil alters the osmotic balance between roots and soil solution, imposing drought-like conditions for plants [94]. Furthermore, salts accumulation in plants tissues may reach toxic levels, hence having negative impacts on plant growth, leaf expansion, and photosynthetic efficiency [93].

Plants cope with saline conditions through an ensemble on biochemical strategies such as ions exclusion, compartmentalization (both at cellular and whole-plant level), synthesis of compatible solutes, change in photosynthetic pathway, and production of plant hormones as cytokinins and ABA [93]. In particular, ABA seems to play a pivotal role in response coordination under salt stress [95].

On the base of studies investigating salinity effects on plant varieties differing in ABA synthesis and sensitivity, it has been highlighted that plants have a set of genes regulating osmotic stress. The expression of these genes is triggered by ABA, which binds an ABA-response element (ABRE). Then, the accumulation of Ca^2+^ ions is induced in cytosol, which (coupled with reactive oxygen species under severe stress) act as secondary messengers triggering salt-response genes expression [9,95]. Effects related to ABA are many and include production of compatible osmolytes and antioxidants [9], Ca^2+^ uptake to maintain membranes’ stability [96], reduced induction of leaf abscission by downregulating ethylene release and toxic Cl^−^ ions in leaves [97], change of membranes composition [9], and stomatal closure [95]. Moreover, high [ABA] during drought stress has been demonstrated to counteract the salt-induced photosynthesis downregulation [98], promote starch degradation, and coordinate carbohydrates mobilization [10].

In grapevine, salinity effects have been mainly investigated in arid or semiarid regions (e.g., Australia), where soils are naturally more saline, hence affecting vineyard cropping [99,100,101]. Despite, in the recent years, the interest in this topic has increased worldwide [102,103,104], studies investigating salt-stress responses in grapevine rather focus on plant performance in terms of fruit quality [105,106]. In fact, it has been demonstrated that the excess of salts causes accumulation of Cl^−^ and Na^+^ ions in berry juice, negatively affecting fruit yield, berry quality, and wine production [99,100,107,108].

A recent study investigated the synergic effect of the partial root drying (PRD) irrigation techniques and moderate salinity conditions on Shiraz and Grenache grapevines, in order to evaluate whether PRD-induced stomatal closure may limit xylem loading of toxic ions [109]. Their results assessed that under PRD irrigation regime, vines were generally enriched in Na^+^, K^+^, Cl^−^, and Ca^2+^ ions, if compared to well-watered (WW) plants with same salt conditions. However, in PRD vines Cl^−^ concentration was minor in leaves and higher in roots. These results combined with the lower roots biomass production under WW conditions, confirm previous observations claiming the root system being the pivotal site for ion-exclusion mechanisms [107,108,109,110]. Indeed, it has been demonstrated that salt uptake and translocation is regulated differently in different rootstocks, and the whole process depends on many mechanisms [111]. In 2010, Upreti and Murti [110] reported that under salt stress, more tolerant rootstocks accumulate more ABA. Despite these aspects have been poorly investigated, there are some evidences claiming that grapevine cultivars differing in anisohydric behavior differ in uptake and translocation of toxic ions [107,109,111,112].

Nowadays, literature focusing on ABA-mediated responses under salt stress is still scarce. However, the reported studies confirm the evidences on other species suggesting ABA being a pivotal hormone in salt-stress response mediation [113]. Hence, further investigations on this topic will be useful to better understand the underrated ABA role in this process.

## 6. Conclusions

ABA was widely investigated in the last decades due its role in regulating several physiological processes, including abiotic stress responses. While the understanding of biosynthetic pathway is consolidated, the triggering system of ABA signaling is still debated. In grapevine, thought hypothesis of ABA being mainly synthetized in the root system has been commonly accepted, a growing body of literature has been supporting the hypothesis that drought-induced stomatal closure is mediated by ABA produced into the leaf.

Another debated point is the ABA role at different intensities of stress: while there is consensus in the role of ABA in keeping stomata closed in order to inhibit premature stomata reopening after the end of water stress, its role in regulating the stomatal conductance at the onset of the stress is quite debated. Recent progress in the determination of ABA biosynthesis triggering mechanisms support the hypothesis of primary stomatal closure being induced by an ensemble of passive and active mechanisms unrelated to ABA, which seems to be mainly involved in more severe water stress response.

The dispute on the role of ABA in stomata regulation also involves the debate on the classification of grapevine genotypes in anisohydric and near-anisohydric categories. Investigations carried out in *V. vinifera* and other species have led to the conclusion that genotypes are not divided in two different categories but are distributed along a continuum, according to their degree of anisohydry. The physiological explanation of the different levels of anisohydry lies on the unraveling of the relative importance of the mechanisms inducing stomata regulation in response to the various environmental stimuli. Nevertheless, the attempt of phenotyping the genotypic response to drought is increasing in the last years, and it will provide useful information for viticulture as well as for the comprehension of grapevine physiology. An interesting topic in future research may involve the investigation of differences in the basal level of ABA and modulations of ABA content, in varieties differing for abiotic stress tolerance.

Although, in the last decades, the understanding of the physiological processes in which ABA is involved has notably increased, many aspects are still debated. In *V. vinifera*, future research efforts should be aimed at the comprehension of the role of ABA in drought stress in relation to water stress severity, duration, and frequency in order to make the experimental results more representative to what occurs in the field. Emerging topics deserving more attention are the interaction of ABA regulation and carbohydrates under water stress and the role of ABA in salt stress response, which is still poorly investigated, especially in this species.

## References

[1] Choat B., Jansen S., Brodribb T.J., Cochard H., Delzon S., Bhaskar R., Bucci S.J., Feild T.S., Gleason S.M., Hacke U.G. (2012). Global convergence in the vulnerability of forests to drought. Nature.

[2] Stocker T.F., Qin D., Plattner G.-K., Tignor M., Allen S.K., Boschung J., Nauels A., Xia Y., Bex V., Midgley P.M. (2013). Climate Change 2013: The Physical Science Basis.

[3] Costa J.M., Vaz M., Escalona J., Egipto R., Lopes C., Medrano H., Chaves M.M. (2016). Modern viticulture in southern Europe: Vulnerabilities and strategies for adaptation to water scarcity. Agric. Water Manag..

[4] Duchêne E., Huard F., Dumas V., Schneider C., Merdinoglu D. (2010). The challenge of adapting grapevine varieties to climate change. Clim. Res..

[5] Gambetta G.A., Herrera J.C., Dayer S., Feng Q., Hochberg U., Castellarin S.D. (2020). The physiology of drought stress in grapevine: Towards an integrative definition of drought tolerance. J. Exp. Bot..

[6] Perrone I., Pagliarani C., Lovisolo C., Chitarra W., Roman F., Schubert A. (2012). Recovery from water stress affects grape leaf petiole transcriptome. Planta.

[7] Savi T., Casolo V., Dal Borgo A., Rosner S., Torboli V., Stenni B., Bertoncin P., Martellos S., Pallavicini A., Nardini A. (2019). Drought-induced dieback of *Pinus nigra*: A tale of hydraulic failure and carbon starvation. Conserv. Physiol..

[8] Lovisolo C., Perrone I., Carra A., Ferrandino A., Flexas J., Medrano H., Schubert A. (2010). Drought-induced changes in development and function of grapevine (*Vitis* spp.) organs and in their hydraulic and non-hydraulic interactions at the whole-plant level: A physiological and molecular update. Funct. Plant Biol..

[9] Vishwakarma K., Upadhyay N., Kumar N., Yadav G., Singh J., Mishra R.K., Kumar V., Verma R., Upadhyay R.G., Pandey M. (2017). Abscisic Acid Signaling and Abiotic Stress Tolerance in Plants: A Review on Current Knowledge and Future Prospects. Front. Plant Sci..

[10] Kempa S., Krasensky J., Santo S.D., Kopka J., Jonak C. (2008). A Central Role of Abscisic Acid in Stress-Regulated Carbohydrate Metabolism. PLoS ONE.

[11] Millar A.A., Jacobsen J.V., Ross J.J., Helliwell C.A., Poole A.T., Scofield G., Reid J.B., Gubler F. (2006). Seed dormancy and ABA metabolism in Arabidopsis and barley: The role of ABA 8′-hydroxylase. Plant J..

[12] Ding Q., Zeng J., He X.-Q. (2016). MiR169 and its target PagHAP2-6 regulated by ABA are involved in poplar cambium dormancy. J. Plant Physiol..

[13] De Smet I., Signora L., Beeckman T., Inzé D., Foyer C.H., Zhang H. (2003). An abscisic acid-sensitive checkpoint in lateral root development of *Arabidopsis*. Plant J..

[14] Jia H.-F., Chai Y.-M., Li C.-L., Lu D., Luo J.-J., Qin L., Shen Y.-Y. (2011). Abscisic Acid Plays an Important Role in the Regulation of Strawberry Fruit Ripening. Plant Physiol..

[15] Downton W.J.S., Loveys B.R., Grant W.J.R. (1990). Salinity effects on the stomatal behaviour of grapevine. New Phytol..

[16] Dodd I.C., Egea G., Davies W.J. (2008). Abscisic acid signalling when soil moisture is heterogeneous: Decreased photoperiod sap flow from drying roots limits abscisic acid export to the shoots. Plant Cell Environ..

[17] Dodd I.C., Puértolas J., Huber K., Pérez-Pérez J.G., Wright H.R., Blackwell M.S. (2015). The importance of soil drying and re-wetting in crop phytohormonal and nutritional responses to deficit irrigation. J. Exp. Bot..

[18] Frioni T., Tombesi S., Sabbatini P., Squeri C., Lavado Rodas N., Palliotti A., Poni S. (2020). Kaolin Reduces ABA Biosynthesis through the Inhibition of Neoxanthin Synthesis in Grapevines under Water Deficit. Int. J. Mol. Sci..

[19] Tombesi S., Nardini A., Frioni T., Soccolini M., Zadra C., Farinelli D., Poni S., Palliotti A. (2015). Stomatal closure is induced by hydraulic signals and maintained by ABA in drought-stressed grapevine. Sci. Rep..

[20] Falchi R., Petrussa E., Braidot E., Sivilotti P., Boscutti F., Vuerich M., Calligaro C., Filippi A., Herrera J.C., Sabbatini P. (2020). Analysis of Non-Structural Carbohydrates and Xylem Anatomy of Leaf Petioles Offers New Insights in the Drought Response of Two Grapevine Cultivars. Int. J. Mol. Sci..

[21] Saleh B., Alshehadah E., Slaman H. (2020). Abscisic Acid (ABA) and Salicylic Acid (SA) Content in Relation to Transcriptional Patterns in Grapevine (*Vitis vinifera* L.) under Salt Stress. J. Plant Biochem. Physiol..

[22] Banerjee A., Sharkey T.D. (2014). Methylerythritol 4-phosphate (MEP) pathway metabolic regulation. Nat. Prod. Rep..

[23] Loveys B.R., Kriedemann P.E. (1974). Internal Control of Stomatal Physiology and Photosynthesis. I. Stomatal Regulation and Associated Changes in Endogenous Levels of Abscisic and Phaseic Acids. Funct. Plant Biol..

[24] Loveys B.R. (1977). The Intracellular Location of Abscisic Acid in Stressed and Non-Stressed Leaf Tissue. Physiol. Plant..

[25] Loveys B.R., Düring H. (1984). Diurnal Changes in Water Relations and Abscisic Acid in Field-Grown *Vitis Vinifera* Cultivars. New Phytol..

[26] Pierce M., Raschke K. (1980). Correlation between loss of turgor and accumulation of abscisic acid in detached leaves. Planta.

[27] Zhang J., Davies W.J. (1987). Increased Synthesis of ABA in Partially Dehydrated Root Tips and ABA Transport from Roots to Leaves. J. Exp. Bot..

[28] Zhang J., Davies W.J. (1990). Changes in the concentration of ABA in xylem sap as a function of changing soil water status can account for changes in leaf conductance and growth. Plant Cell Environ..

[29] Soar C.J., Dry P.R., Loveys B.R. (2006). Scion photosynthesis and leaf gas exchange in *Vitis vinifera* L. cv. Shiraz: Mediation of rootstock effects via xylem sap ABA. Aust. J. Grape Wine Res..

[30] Speirs J., Binney A., Collins M., Edwards E., Loveys B. (2013). Expression of ABA synthesis and metabolism genes under different irrigation strategies and atmospheric VPDs is associated with stomatal conductance in grapevine (*Vitis vinifera* L. cv Cabernet Sauvignon). J. Exp. Bot..

[31] Dry P.R., Loveys B.R., During H. (2000). Partial drying of the rootzone of grape I. Transient changes in shoot growth and gas exchange. Vitis-Geilweilerhof-.

[32] Poni S., Bernizzoni F., Civardi S. (2007). Response of “Sangiovese” grapevines to partial root-zone drying: Gas-exchange, growth and grape composition. Sci. Hortic..

[33] Romero P., Dodd I.C., Martinez-Cutillas A. (2012). Contrasting physiological effects of partial root zone drying in field-grown grapevine (*Vitis vinifera* L. cv. Monastrell) according to total soil water availability. J. Exp. Bot..

[34] Romero P., Pérez-Pérez J.G., Amor F.M.D., Martinez-Cutillas A., Dodd I.C., Botía P. (2014). Partial root zone drying exerts different physiological responses on field-grown grapevine (*Vitis vinifera* cv. Monastrell) in comparison to regulated deficit irrigation. Funct. Plant Biol..

[35] Schachtman D.P., Goodger J.Q.D. (2008). Chemical root to shoot signaling under drought. Trends Plant Sci..

[36] Dodd I.C., Theobald J.C., Bacon M.A., Davies W.J. (2006). Alternation of wet and dry sides during partial rootzone drying irrigation alters root-to-shoot signalling of abscisic acid. Funct. Plant Biol..

[37] Kochhar S.L., Gujral S.K. (2020). Plant Physiology: Theory and Applications: Theory and Applications.

[38] Pérez-Pérez J.G., Puertolas J., Albacete A., Dodd I.C. (2020). Alternation of wet and dry sides during partial rootzone drying irrigation enhances leaf ethylene evolution. Environ. Exp. Bot..

[39] Soar C.J., Speirs J., Maffei S.M., Penrose A.B., Mccarthy M.G., Loveys B.R. (2006). Grape vine varieties Shiraz and Grenache differ in their stomatal response to VPD: Apparent links with ABA physiology and gene expression in leaf tissue. Aust. J. Grape Wine Res..

[40] Ikegami K., Okamoto M., Seo M., Koshiba T. (2008). Activation of abscisic acid biosynthesis in the leaves of *Arabidopsis thaliana* in response to water deficit. J. Plant Res..

[41] Manzi M., Lado J., Rodrigo M.J., Zacarías L., Arbona V., Gómez-Cadenas A. (2015). Root ABA Accumulation in Long-Term Water-Stressed Plants is Sustained by Hormone Transport from Aerial Organs. Plant Cell Physiol..

[42] McAdam S.A.M., Brodribb T.J. (2018). Mesophyll Cells Are the Main Site of Abscisic Acid Biosynthesis in Water-Stressed Leaves. Plant Physiol..

[43] Sack L., John G.P., Buckley T.N. (2018). ABA Accumulation in Dehydrating Leaves Is Associated with Decline in Cell Volume, Not Turgor Pressure. Plant Physiol..

[44] Castro P., Puertolas J., Dodd I.C. (2019). Stem girdling uncouples soybean stomatal conductance from leaf water potential by enhancing leaf xylem ABA concentration. Environ. Exp. Bot..

[45] Li L., Yuan H. (2013). Chromoplast biogenesis and carotenoid accumulation. Arch. Biochem. Biophys..

[46] Howitt C.A., Pogson B.J. (2006). Carotenoid accumulation and function in seeds and non-green tissues. Plant Cell Environ..

[47] Kuromori T., Fujita M., Urano K., Tanabata T., Sugimoto E., Shinozaki K. (2016). Overexpression of AtABCG25 enhances the abscisic acid signal in guard cells and improves plant water use efficiency. Plant Sci..

[48] Bota B.J., Flexas J., Medrano H. (2001). Genetic variability of photosynthesis and water use in Balearic grapevine cultivars. Ann. Appl. Biol..

[49] Levin A.D., Williams L.E., Matthews M.A. (2020). A continuum of stomatal responses to water deficits among 17 wine grape cultivars (*Vitis vinifera*). Funct. Plant Biol..

[50] Pagliarani C., Vitali M., Ferrero M., Vitulo N., Incarbone M., Lovisolo C., Valle G., Schubert A. (2017). The Accumulation of miRNAs Differentially Modulated by Drought Stress Is Affected by Grafting in Grapevine. Plant Physiol..

[51] Bota J., Tomás M., Flexas J., Medrano H., Escalona J.M. (2016). Differences among grapevine cultivars in their stomatal behavior and water use efficiency under progressive water stress. Agric. Water Manag..

[52] Tyree M.T. (1997). The Cohesion-Tension theory of sap ascent: Current controversies. J. Exp. Bot..

[53] Tyree M.T., Sperry J.S. (1989). Vulnerability of Xylem to Cavitation and Embolism. Annu. Rev. Plant Physiol. Plant Mol. Biol..

[54] Hacke U.G., Sperry J.S., Pockman W.T., Davis S.D., McCulloh K.A. (2001). Trends in wood density and structure are linked to prevention of xylem implosion by negative pressure. Oecologia.

[55] Buckley T.N. (2019). How do stomata respond to water status?. New Phytol..

[56] Mäser P., Leonhardt N., Schroeder J. (2003). The Clickable Guard Cell: Electronically Linked Model of Guard Cell Signal Transduction Pathways. Arab. Book.

[57] Wang Y., Chen Z.-H., Zhang B., Hills A., Blatt M.R. (2013). PYR/PYL/RCAR Abscisic Acid Receptors Regulate K^+^ and Cl^-^ Channels through Reactive Oxygen Species-Mediated Activation of Ca^2+^ Channels at the Plasma Membrane of Intact Arabidopsis Guard Cells. Plant Physiol..

[58] Tardieu F., Simonneau T. (1998). Variability among species of stomatal control under fluctuating soil water status and evaporative demand: Modelling isohydric and anisohydric behaviours. J. Exp. Bot..

[59] Ratzmann G., Meinzer F.C., Tietjen B. (2019). Iso/Anisohydry: Still a Useful Concept. Trends Plant Sci..

[60] Hochberg U., Rockwell F.E., Holbrook N.M., Cochard H. (2018). Iso/Anisohydry: A Plant–Environment Interaction Rather Than a Simple Hydraulic Trait. Trends Plant Sci..

[61] Coupel-Ledru A., Lebon É., Christophe A., Doligez A., Cabrera-Bosquet L., Péchier P., Hamard P., This P., Simonneau T. (2014). Genetic variation in a grapevine progeny (*Vitis vinifera* L. cvs Grenache × Syrah) reveals inconsistencies between maintenance of daytime leaf water potential and response of transpiration rate under drought. J. Exp. Bot..

[62] Nardini A., Salleo S. (2000). Limitation of stomatal conductance by hydraulic traits: Sensing or preventing xylem cavitation?. Trees.

[63] Christmann A., Weiler E.W., Steudle E., Grill E. (2007). A hydraulic signal in root-to-shoot signalling of water shortage. Plant J..

[64] Hubbard R.M., Bond B.J., Ryan M.G. (1999). Evidence that hydraulic conductance limits photosynthesis in old *Pinus ponderosa* trees. Tree Physiol..

[65] Klein T. (2014). The variability of stomatal sensitivity to leaf water potential across tree species indicates a continuum between isohydric and anisohydric behaviours. Funct. Ecol..

[66] Tombesi S., Nardini A., Farinelli D., Palliotti A. (2014). Relationships between stomatal behavior, xylem vulnerability to cavitation and leaf water relations in two cultivars of *Vitis vinifera*. Physiol. Plant..

[67] Skelton R.P., West A.G., Dawson T.E. (2015). Predicting plant vulnerability to drought in biodiverse regions using functional traits. Proc. Natl. Acad. Sci. USA.

[68] Hochberg U., Windt C.W., Ponomarenko A., Zhang Y.-J., Gersony J., Rockwell F.E., Holbrook N.M. (2017). Stomatal Closure, Basal Leaf Embolism, and Shedding Protect the Hydraulic Integrity of Grape Stems. Plant Physiol..

[69] McAdam S.A.M., Brodribb T.J. (2014). Separating Active and Passive Influences on Stomatal Control of Transpiration. Plant Physiol..

[70] Degu A., Hochberg U., Wong D.C., Alberti G., Lazarovitch N., Peterlunger E., Castellarin S.D., Herrera J.C., Fait A. (2019). Swift metabolite changes and leaf shedding are milestones in the acclimation process of grapevine under prolonged water stress. BMC Plant Biol..

[71] Tramontini S., Döring J., Vitali M., Ferrandino A., Stoll M., Lovisolo C. (2014). Soil water-holding capacity mediates hydraulic and hormonal signals of near-isohydric and near-anisohydric *Vitis* cultivars in potted grapevines. Funct. Plant Biol..

[72] Coupel-Ledru A., Tyerman S.D., Masclef D., Lebon E., Christophe A., Edwards E.J., Simonneau T. (2017). Abscisic acid down-regulates hydraulic conductance of grapevine leaves in isohydric genotypes only. Plant Physiol..

[73] Dayer S., Scharwies J.D., Ramesh S.A., Sullivan W., Doerflinger F.C., Pagay V., Tyerman S.D. (2020). Comparing hydraulics between two grapevine cultivars reveals differences in stomatal regulation under water stress and exogenous ABA applications. Front. Plant Sci..

[74] Gambetta G.A., Knipfer T., Fricke W., McElrone A.J., Chaumont F., Tyerman S.D. (2017). Aquaporins and Root Water Uptake. Plant Aquaporins: From Transport to Signaling.

[75] Dayer S., Reingwirtz I., McElrone A.J., Gambetta G.A. (2019). Response and Recovery of Grapevine to Water Deficit: From Genes to Physiology. The Grape Genome.

[76] McDowell N.G., Beerling D.J., Breshears D.D., Fisher R.A., Raffa K.F., Stitt M. (2011). The interdependence of mechanisms underlying climate-driven vegetation mortality. Trends Ecol. Evol..

[77] Tomasella M., Petrussa E., Petruzzellis F., Nardini A., Casolo V. (2020). The Possible Role of Non-Structural Carbohydrates in the Regulation of Tree Hydraulics. Int. J. Mol. Sci..

[78] Secchi F., Pagliarani C., Cavalletto S., Petruzzellis F., Tonel G., Savi T., Tromba G., Obertino M.M., Lovisolo C., Nardini A. (2020). Chemical inhibition of xylem cellular activity impedes the removal of drought-induced embolisms in poplar stems—New insights from micro-CT analysis. New Phytol..

[79] Brodersen C.R., McElrone A.J., Choat B., Lee E.F., Shackel K.A., Matthews M.A. (2013). In vivo visualizations of drought-induced embolism spread in *Vitis vinifera*. Plant Physiol..

[80] Pan Q.-H., Li M.-J., Peng C.-C., Zhang N., Zou X., Zou K.-Q., Wang X.-L., Yu X.-C., Wang X.-F., Zhang D.-P. (2005). Abscisic acid activates acid invertases in developing grape berry. Physiol. Plant..

[81] Thalmann M., Pazmino D., Seung D., Horrer D., Nigro A., Meier T., Kölling K., Pfeifhofer H.W., Zeeman S.C., Santelia D. (2016). Regulation of Leaf Starch Degradation by Abscisic Acid Is Important for Osmotic Stress Tolerance in Plants. Plant Cell.

[82] Secchi F., Perrone I., Chitarra W., Zwieniecka A.K., Lovisolo C., Zwieniecki M.A. (2013). The Dynamics of Embolism Refilling in Abscisic Acid (ABA)-Deficient Tomato Plants. Int. J. Mol. Sci..

[83] Brunetti C., Savi T., Nardini A., Loreto F., Gori A., Centritto M. (2020). Changes in abscisic acid content during and after drought are related to carbohydrate mobilization and hydraulic recovery in poplar stems. Tree Physiol..

[84] Cardoso A.A., Gori A., Da-Silva C.J., Brunetti C. (2020). Abscisic Acid Biosynthesis and Signaling in Plants: Key Targets to Improve Water Use Efficiency and Drought Tolerance. Appl. Sci..

[85] Tomasella M., Häberle K.-H., Nardini A., Hesse B., Machlet A., Matyssek R. (2017). Post-drought hydraulic recovery is accompanied by non-structural carbohydrate depletion in the stem wood of Norway spruce saplings. Sci. Rep..

[86] Tortosa I., Escalona J.M., Douthe C., Pou A., Garcia-Escudero E., Toro G., Medrano H. (2019). The intra-cultivar variability on water use efficiency at different water status as a target selection in grapevine: Influence of ambient and genotype. Agric. Water Manag..

[87] Rogiers S.Y., Hardie W.J., Smith J.P. (2011). Stomatal density of grapevine leaves (*Vitis vinifera* L.) responds to soil temperature and atmospheric carbon dioxide. Aust. J. Grape Wine Res..

[88] Walter J., Nagy L., Hein R., Rascher U., Beierkuhnlein C., Willner E., Jentsch A. (2011). Do plants remember drought? Hints towards a drought-memory in grasses. Environ. Exp. Bot..

[89] Lukić N., Kukavica B., Davidović-Plavšić B., Hasanagić D., Walter J. (2020). Plant stress memory is linked to high levels of anti-oxidative enzymes over several weeks. Environ. Exp. Bot..

[90] Li Y., Xu S., Wang Z., He L., Xu K., Wang G. (2018). Glucose triggers stomatal closure mediated by basal signaling through HXK1 and PYR/RCAR receptors in Arabidopsis. J. Exp. Bot..

[91] Nguyen T.T.Q., Trinh L.T.H., Pham H.B.V., Le T.V., Phung T.K.H., Lee S.-H., Cheong J.-J. (2020). Evaluation of proline, soluble sugar and ABA content in soybean *Glycine max* (L.) under drought stress memory. AIMS Bioeng..

[92] Netzer Y., Munitz S., Shtein I., Schwartz A. (2019). Structural memory in grapevines: Early season water availability affects late season drought stress severity. Eur. J. Agron..

[93] Parida A.K., Das A.B. (2005). Salt tolerance and salinity effects on plants: A review. Ecotoxicol. Environ. Saf..

[94] Laurenson S., Bolan N.S., Smith E., Mccarthy M. (2012). Review: Use of recycled wastewater for irrigating grapevines. Aust. J. Grape Wine Res..

[95] Zhang J., Jia W., Yang J., Ismail A.M. (2006). Role of ABA in integrating plant responses to drought and salt stresses. Field Crop. Res..

[96] Chen S., Li J., Wang S., Hüttermann A., Altman A. (2001). Salt, nutrient uptake and transport, and ABA of *Populus euphratica*; a hybrid in response to increasing soil NaCl. Trees.

[97] Gomez-Cadenas A., Arbona V., Jacas J., Primo-Millo E., Talon M. (2002). Abscisic acid reduces leaf abscission and increases salt tolerance in *citrus* plants. J. Plant Growth Regul..

[98] Popova L.P., Stoinova Z.G., Maslenkova L.T. (1995). Involvement of abscisic acid in photosynthetic process in *Hordeum vulgare* L. during salinity stress. J. Plant Growth Regul..

[99] Downton W.J.S. (1977). Influence of rootstocks on the accumulation of chloride, sodium and potassium in grapevines. Aust. J. Agric. Res..

[100] Stevens R.M., Harvey G., Partington D.L. (2011). Irrigation of grapevines with saline water at different growth stages: Effects on leaf, wood and juice composition. Aust. J. Grape Wine Res..

[101] Walker R.R., Blackmore D.H., Clingeleffer P.R., Emanuelli D. (2014). Rootstock type determines tolerance of Chardonnay and Shiraz to long-term saline irrigation. Aust. J. Grape Wine Res..

[102] Hassena A.B., Zouari M., Trabelsi L., Khabou W., Zouari N. (2018). Physiological improvements of young olive tree (*Olea europaea* L. cv. Chetoui) under short term irrigation with treated wastewater. Agric. Water Manag..

[103] Martin L., Vila H., Bottini R., Berli F. (2020). Rootstocks increase grapevine tolerance to NaCl through ion compartmentalization and exclusion. Acta Physiol. Plant..

[104] Aydemir B.Ç., Özmen C.Y., Kibar U., Mutaf F., Büyük P.B., Bakır M., Ergül A. (2020). Salt stress induces endoplasmic reticulum stress-responsive genes in a grapevine rootstock. PLoS ONE.

[105] Li F., Zhao X., Yang X., He X. (2020). Influence of NaCl stress on ABA synthesis and hardness of *Vitis vinifera* L.‘Cabernet Sauvignon’ berry in veraison. Proceedings of the AIP Conference Proceedings, Proceedings of the 2nd International Conference on Frontiers of Biological Sciences and Engineering (FBSE 2019).

[106] Zhao P., Yang X., Han N., He X. (2020). Influence of salt-alkali stress on quality formation of *Vitis vinifera* L.‘Cabernet Sauvignon’ of wine grape. Proceedings of the AIP Conference Proceedings, Proceedings of the 2nd International Conference on Frontiers of Biological Sciences and Engineering (FBSE 2019).

[107] Walker R.R., Read P.E., Blackmore D.H. (2000). Rootstock and salinity effects on rates of berry maturation, ion accumulation and colour development in Shiraz grapes. Aust. J. Grape Wine Res..

[108] Teakle N.L., Tyerman S.D. (2010). Mechanisms of Cl^-^ transport contributing to salt tolerance. Plant Cell Environ..

[109] Degaris K.A., Walker R.R., Loveys B.R., Tyerman S.D. (2016). Comparative effects of deficit and partial root-zone drying irrigation techniques using moderately saline water on ion partitioning in Shiraz and Grenache grapevines. Aust. J. Grape Wine Res..

[110] Upreti K.K., Murti G.S.R. (2010). Response of grape rootstocks to salinity: Changes in root growth, polyamines and abscisic acid. Biol. Plant..

[111] Paranychianakis N.V., Angelakis A.N. (2008). The effect of water stress and rootstock on the development of leaf injuries in grapevines irrigated with saline effluent. Agric. Water Manag..

[112] Vincent D., Ergül A., Bohlman M.C., Tattersall E.A.R., Tillett R.L., Wheatley M.D., Woolsey R., Quilici D.R., Joets J., Schlauch K. (2007). Proteomic analysis reveals differences between *Vitis vinifera* L. cv. Chardonnay and cv. Cabernet Sauvignon and their responses to water deficit and salinity. J. Exp. Bot..

[113] Pye M.F., Dye S.M., Resende R.S., MacDonald J.D., Bostock R.M. (2018). Abscisic acid as a dominant signal in tomato during salt stress predisposition to phytophthora root and crown rot. Front. Plant Sci..

